# Bovine *Staphylococcus aureus*: a European study of contagiousness and antimicrobial resistance

**DOI:** 10.3389/fvets.2023.1154550

**Published:** 2023-05-03

**Authors:** Ghazal Nemati, Alicia Romanó, Fabian Wahl, Thomas Berger, Laura Vazquez Rojo, Hans Ulrich Graber

**Affiliations:** ^1^Food Microbial Systems, Risk Assessment and Mitigation Group, Agroscope, Bern, Switzerland; ^2^Food Microbial Systems, Microbiological Safety of Foods of Animal Origin Group, Agroscope, Bern, Switzerland; ^3^Food Microbial Systems, Agroscope, Bern, Switzerland

**Keywords:** *Staphylococcus aureus*, *adlb*, antimicrobial resistance, minimum inhibitory concentration, multidrug resistant

## Abstract

In dairy herds managements, mastitis is the leading cause of economic losses. One of the most important pathogens responsible for intra-mammary infections is *Staphylococcus aureus*. The genetic properties of *S. aureus* have a strong influence on its pathogenicity and contagiousness. In this study, we aimed to obtain a comprehensive overview of the key bovine *S. aureus* clinical properties, such as contagiousness and antimicrobial resistance, present in European strains. For this, 211 bovine *S. aureus* strains from ten European countries that were used in a previous study were used in this study. Contagiousness was assessed using qPCR for the detection of the marker gene *adlb*. Antimicrobial resistance was evaluated using a broth microdilution assay and mPCR for the detection of genes involved in penicillin resistance (*blaI, blaR1*, and *blaZ*). It was found that *adlb* was present in CC8/CLB strains; however, in Germany, it was found in CC97/CLI and in an unknown CC/CLR strains. CC705/CLC strains from all countries were found to be susceptible to all tested antibiotics. Major resistance to penicillin/ampicillin, chloramphenicol, clindamycin and tetracycline was detected. Resistance to oxacillin, trimethoprim/sulfamethoxazole and cephalosporins was rarely observed. In addition, contagiousness and antibiotic resistance seem to correlate with different CCs and genotypic clusters. Hence, it is recommended that multilocus sequence typing or genotyping be utilized as a clinical instrument to identify the most appropriate antibiotic to use in mastitis treatment. Actualization of the breakpoints of veterinary strains is necessary to address the existing antibiotic resistance of the bacteria involved in veterinary mastitis.

## 1. Introduction

In veterinary medicine, mastitis is the leading cause of economic losses in dairy herds management. It contributes to reductions in milk quality and production, there are costs associated with its treatment, and animal culling can be a consequence of treatment failures (1, 2). In Switzerland, the total cost of mastitis is ~$131 million annually, according to Heiniger et al. (3). One of the most important pathogens responsible for intramammary infections (IMIs) is *Staphylococcus aureus* (4). *S. aureus* may infect only some individual animals or may be contagious and infect the entire herd; infections usually resulting in subclinical chronic mastitis (5, 6). As shown previously (5, 7–12), the genetic properties of *S. aureus* have a strong influence on its pathogenicity and contagiousness, making subtyping necessary to improve treatment success and dairy herd management. Using ribosomal spacer PCR (RS-PCR), it has been shown that the rate of infected cows in a herd is highly dependent on the bacterial genotype (GT) (7–10), and, *S. aureus* genotype B (GTB) and its variants may infect up to 100% of cows in the same herd (7–9, 11) due to its high contagiousness (13, 14). In contrast, other genotypes and their variants (e.g., GTC, GTF, GTS) are restricted to one or a few cows in a herd (7–10, 15, 16). In the electrophoresis of the RS-PCR product, variants differ in 1 electrophoretic band and as consequence, are named by superscripted roman numerals (e.g., GTR^I^). For further simplification, genotypes and their variants are combined into genotypic clusters (CL). For example, GTB and its variants form a cluster named CLB. Multilocus sequence typing (MLST) (17) results have shown that CLB is almost exclusively associated with clonal complex 8 (CC8), whereas CLC corresponds largely to CC705, and CLR to CC97 and CC133 (9, 18). In Europe, CLB, CLC, CLF, CLI, and CLR account for 76.6% of all *S. aureus* isolates obtained from clinical milk samples (19).

RS-PCR is particularly suitable for clinical applications as it is a low-cost, high-throughput method that provides analytical resolution at least as good as *spa* typing in bovine strains (9, 18). However, it is more appropriate to use MLST for subtyping at the biological level because a *S. aureus* clone can be used (17) and, consequently, evolutionary identity established (20, 21). To sanitize Swiss dairy herds infected with the contagious *S. aureus* CLB, Sartori et al., developed a real-time quantitative PCR (qPCR) assay to identify this pathogen in milk samples and achieved diagnostic sensitivity and specificity at the cow level of 99 and 100%, respectively (22). This new assay has been used to detect, with high specificity, the gene *adlb* which encodes the bovine adhesion-like protein located in the GTB-specific staphylococcal cassette chromosome SCCgtb (16, 22). It is a marker for contagiousness and high prevalence of intra-mammary infection (IMI) in dairy herds (11, 16).

Antibiotic (AB) treatment is still one of the most important measures for controlling bovine mastitis (23). However, the frequently unsatisfactory cure rates remain a serious concern, particularly for IMI caused by *S. aureus* (6, 24–27). One major reason for this drawback is the improper use of ABs (28, 29). Additionally, AB treatments applied at the herd level are usually not reported, even though various mastitis control plans strongly recommend performing these analyses and collecting the resultant data (30). Since 2019, it has been required for Swiss's farms to declare the AB treatments used at the herd level (31). In terms of the ABs used to treatment bovine IMIs caused by *S. aureus*, various classes of AB are used: typically, ß-lactams (penicillins and cephalosporins), aminoglycosides, lincosamides, and macrolides (32, 33). Penicillin G is the most commonly used AB for treating IMI in cows caused by *S. aureus* and other Gram-positive mastitis pathogens. In *S. aureus*, the *bla* operon mediates AB resistance against penicillin G and other β-lactamase-sensitive penicillins. The *bla* operon can be located on plasmids (as transposon) or on the chromosome (34, 35) and contains three genes: (1) *blaZ*, which encodes the βlactamase that hydrolyzes the β-lactam ring of AB, rending them inactive; (2) *blaI*, which encodes the repressor; (3) *blaR1* which encodes the sensor and antirepressor (35, 36). Ivanovic et al., recently showed that the *bla* operon plays a key role in phenotypic resistance to penicillin. Furthermore, for *S. aureus*, they highlighted the importance of using the minimum inhibitory concentration (MIC) value as the gold standard when assessing resistance to penicillin and probably other ABs (33).

As contagiousness and antimicrobial resistance (AMR) are critical pathogenic factors of the *S. aureus* strains responsible for bovine mastitis, a comprehensive study was performed to assess the distribution of these key clinical properties in strains from across Europe. Contagiousness was assessed using qPCR to detect the *adlb* gene, which is a staphylococcal marker for contagiousness and for high prevalence of intra-mammary infection in dairy herds. Furthermore, AMR was evaluated using an MIC assay and melting curve PCR (mPCR) to detect genes involved in penicillin resistance (*blaI, blaR1*, and *blaZ*).

## 2. Materials and methods

### 2.1. Strain collection

A total of 211 bovine strains of *S. aureus* were used in this study that had been collected from 10 European countries; Austria, Belgium, France, Germany, Ireland, Italy, Macedonia, Norway, Slovenia, and Switzerland. These strains were originally collected during two previous studies by Boss et al. (18) and Cosandey et al. (19). As described by Cosandey et al., the strains were aseptically collected from milk samples from individual quarters (19). The strains had been stored in skim milk at −20°C. They were plated onto Columbia agar plates containing 5% sheep blood (Biomérieux Suisse s.a., Geneva, Switzerland) and incubated at 37°C for 24 h (18, 19). The genotypes (GT) and the clonal complexes (CCs) information was obtained from previous studies. The distribution of the different CCs and the GT across the 10 European countries is shown in Table 1 (19).

**Table 1:** Distribution of *Staphylococcus aureus* genotypes and clonal complexes across 10 European countries.

	**Clonal complex (CC)**	**Genotype (GT)**
Austria	CC8 (9)	GTB (5), GTAM (2), GTI^IV^ (1), GTBE (1)
	CC97 (10)	GTR (2), GTR^I^ (1), GTR^VI^ (3), GTBC (1), GTBL (2), GTE (1)
	CC705 (10)	GTC (7), GTC^I^ (1), GTR^VI^ (1), GTZ (1)
	CC20 (1)	GTF (1)
	CC9 (5)	GTF^III^ (4), GTR^VI^ (1)
	Other CC (13)	
	CC5 (1)	GTE (1)
	CC25 (1)	GTAK (1)
	CC30 (1)	GTB (1)
	CC71 (3)	GTR (2), GTR^X^ (1)
	CC101 (3)	GTAH (2), GTR^VII^ (1)
	CC133 (2)	GTR^I^ (1), GTR^VI^ (1)
	CC479 (1)	GTC (1)
	Unknown (1)	GTAH (1)
Belgium	CC8 (1)	GTB^II^ (1)
	CC97 (4)	GTI^I^ (4)
	CC705 (7)	GTC (4), GTC^I^ (2), GTC^II^ (1)
	CC20 (1)	GTF (1)
	Other CC (5)	
	CC70 (1)	GTC^I^ (1)
	CC71 (1)	GTI (1)
	CC133 (2)	GTR (1), GTZ (1)
	CC479 (1)	GTBG (1)
France	CC8 (2)	GTB (2)
	CC705 (3)	GTC (1), GTC^I^ (2)
	CC20 (4)	GTF (4)
	Other CC (2)	
	CC15 (1)	GTJ^I^ (1)
	CC133 (1)	GTR^I^ (1)
Germany	CC8 (11)	GTB (8), GTB^I^ (3)
	CC97 (5)	GTI^I^ (3), GTR^VI^ (2)
	CC705 (3)	GTC (1), GTC^II^ (2)
	CC9 (2)	GTF^III^ (2)
	Other CC (18)	
	CC1 (3)	GTAN (1), GTBA (1), GTBJ (1)
	CC7 (2)	GTL (1), GTM (1)
	CC15 (1)	GTJ (1)
	CC50 (1)	GTAU (1)
	CC71 (1)	GTI (1)
	CC133 (2)	GTR^I^ (1), GTR^II^ (1)
	CC398 (3)	GTS (3)
	CC479 (4)	GTP (1), GTZ (3)
	Unknown (1)	GTR^I^ (1)
Ireland	CC97 (2)	GTR (1), GTR^VI^ (1)
	CC705 (2)	GTC^I^ (1), GTO^I^ (1)
	Other CC (7)	
	CC5 (1)	GTE (1)
	CC71 (6)	GTAN (1), GTR (2), GTR^VI^ (3)
Italy	CC8 (9)	GTB (9)
	CC97 (3)	GTBE^I^ (1), GTF (1), GTI^I^ (1)
	CC20 (1)	GTF (1)
	CC9 (1)	GTF^III^ (1)
	Other CC (6)	
	CC22 (1)	GTP (1)
	CC30 (1)	GTBE^I^ (1)
	CC71 (1)	GTI^I^ (1)
	CC126 (2)	GTS^I^ (2)
	CC398 (1)	GTS (1)
Macedonia	CC97 (1)	GTR^VI^ (1)
	Other CC (2)	
	CC7 (1)	GTM (1)
	Unknown (1)	GTR^VI^ (1)
Norway	CC97 (2)	GTR (2)
	Other CC (4)	
	CC133 (2)	GTZ (2)
	CC479 (1)	GTZ (1)
	Unknown (1)	GTC (1)
Slovenia	CC97 (6)	GTR (1), GTR^II^ (2), GTAA (1), GTO (1), GTZ (1)
	CC20(1)	GTAT (1)
	CC9 (1)	GTBB (1)
	Other CC (5)	
	CC22 (1)	GTI^II^ (1)
	CC49 (2)	GTAA (1), GTR^I^ (1)
	CC101 (1)	GTAA (1)
	CC71 (1)	GTR (1)
Switzerland	CC8 (18)	GTB (18)
	CC97 (1)	GTR (1)
	CC705 (19)	GTC (16), GTC^I^ (1), GTA (1), GTH (1)
	Other CC (4)	
	CC5 (1)	GTE (1)
	CC59 (1)	GTD (1)
	CC70 (1)	GTC (1)
	Unknown (1)	GTC (1)

### 2.2. DNA extraction

DNA was extracted from single *S. aureus* colonies. One colony was picked and resuspended in 100 μL of 10 mM Tris-HCl and 10 mM EDTA (pH = 8.5), incubated at 95°C for 10 min, and immediately placed on ice. The lysates were diluted 1:100 in qPCR H_2_O (SINTETICA S.A, Mendrisio, Switzerland) for use as templates. The samples were stored at −20°C and were analyzed within 2 weeks of extraction (18).

### 2.3. Quantitative PCR (qPCR) with *adlb* and internal control gene

Real-time qPCR was performed with *adlb* and the internal control gene (N gene of canine distemper virus [CDVN]) according to the protocol of Sartori et al. (22). The characteristics of the utilized primers are listed in Supplementary Table S1. DNA amplification was performed using a Magnetic Induction Cycler qPCR real-time thermal cycler (Bio Molecular Systems, Australia) and the following cycling conditions: initial denaturation at 95°C for 3 min followed by 45 running cycles of denaturation at 95°C for 3 s and annealing/elongation at 60°C for 20 s. Two reference strains that were positive for both targets were included as positive controls.

### 2.4. PCR analysis of the *bla* operon genes

The mPCR was performed according to the protocol of Ivanovic et al. (33). Each of the 211 strains was analyzed for the presence of *blaI, blaR1*, and *blaZ;* each gene was detected separately. As per Ivanovic et al., amplicons with a single melting peak identical to the positive control for *blaI, blaR1*, or *blaZ* were considered positive. The characteristics of the utilized primers are listed in Supplementary Table S2.

### 2.5. Assessment of antimicrobial sensitivity

The sensitivity of each strain to 30 antimicrobial agents was tested by minimum inhibitory concentration (MIC) using a PM32 panel (Beckman Coulter, Inc., Brea, CA, USA) following the manufacturer's instructions. The tested ABs concentrations (μg/mL) were as follows: amoxicillin/K clavulanate (0.5/0.25–8/4), ampicillin (0.5–8), azithromycin (1–2), cefepime (4–8), cefotaxime (1–2), cefuroxime (4–8), chloramphenicol (8), ciprofloxacin (0.5–1), clindamycin (0.25–0.5, 2), daptomycin (0.5–4), ertapenem (0.5–1), erythromycin (1–2), fosfomycin (32), fusic acid (2), gentamycin (1–4), imipenem (2–8), levofloxacin (1–2), linezolid (0.5–4), meropenem (2–8), moxifloxacin (0.5–1), nitrofurantoin (64), oxacillin (0.25–2), penicillin (0.03–0.25, 2), rifampin (0.5–2), synercid (1–4), teicoplanin (1–8), tetracycline (1–2), tobramycin (1–4), trimethoprim/sulfamethoxazole (1/19–4/76), and vancomycin (0.25–8). Additionally, cefoxitin (4 μg/mL) screening was performed to determine the presence of methicillin resistant *Staphylococcus aureus* (MRSA) strains. When possible, the current clinical breakpoint of the EUCAST was used (37), otherwise the range specified by the CLSI was applied (38). All the ABs tested and their breakpoints are listed in Supplementary Table S3.

### 2.6. Statistical analysis

Data are expressed as absolute numbers or percentage. To assess the associations among different AB, the corresponding *phi* coefficients were computed and plotted using R 4.0.5 (39) together with the corrplot package v. 0.84. *Phi* values range from −1 to 1 (40). Negative *phi* values indicate a negative, inverse association among both variables, whereas positive *phi* values indicate a positive association. The Kappa test was performed using R 4.0.5 (39) to evaluate the agreement between the MIC and the *bla* mPCR results. Kappa values range from 0 to 1, with values of 0 and 1 indicating no and perfect agreement, respectively (41). To assess penicillin resistance, a loglinear model was computed to analyze the relationships among the factors penicillin, CC, country, and their interactions. The analysis was performed using Systat 13 (Systat Software Inc., Richmond, CA).

## 3. Results

### 3.1. Presence of *adlb* in European *S. aureus* strains

The 211 *S. aureus* strains collected from 10 European countries were assessed using qPCR for the presence of *adlb* and its association with GTs and CCs. Among the 211 strains, 46 were positive for *adlb*. The distribution of *adlb* among the different GTs and CCs and among the 10 European countries is shown in Table 2.

**Table 2:** Detailed distribution of *adlb* across different genotypes and clonal complexes, listed by country.

	**Genotype**	**Clonal complexes**
Austria	GTB (6)	CC8 (5)
		Other CC (1)
Belgium	ND	ND
France	GTB (2)	CC8 (2)
Germany	GTB (10)	CC8 (10)
	GTI (1)	CC97 (1)
	Other GT (1)	Other CC (1)
Ireland	ND	ND
Italy	GTB (8)	CC8 (8)
Macedonia	ND	ND
Norway	ND	ND
Slovenia	ND	ND
Switzerland	GTB (18)	CC8 (18)

ND, Not detected.

An analysis of the GTs found to contain *adlb*, showed that 44 of 47 (94%) CLB strains were positive for *adlb* and that only two strains were positive for *adlb* in the remaining 164 strains (1.2%). Furthermore, the gene was also observed in a German GTI^I^ and a GTR^I^ strain. GTB was not detected in Ireland, Macedonia, Slovenia, or Norway. In Italy, Germany, and Belgium, three GTB strains were found that did not contain *adlb*.

### 3.2. AMR overview in European *S. aureus* strains

An analysis of the MIC data showed that 65% of the strains (*n* = 137) were inhibited by all the tested ABs. Table 3 shows the strains that demonstrated AMR, sorted by CC. Only the ABs to which resistance was exhibited are included.

**Table 3:** Detailed description of the isolates (*n* = 211), their genotypes, and their phenotypic (and mPCR) resistance to the tested antibiotics.

			**Phenotypic results (MIC)**																			**mPCR**
**CCs**	**Country**	**Genotype cluster (CL)**	**GEN^a^**	**TOB^a^**	**CIP^b^**	**LEV^b^**	**MOX^b^**	**TEI^c^**	**CLI^d^**	**AZI^e^**	**ERY^e^**	**AMP^f^**	**OXA^f^**	**PEN^f^**	**TET^g^**	**CHL^h^**	**FOS^h^**	**FUA^h^**	**MOX^h^**	**NIT^h^**	**T/S^h^**	***bla***
CC8 (50)	Austria	CLB (5)																				Pos (4)
		CLI (1)												1								Pos (1)
		CLOG (3)										2		2	1			1				Pos (2)
	Belgium	CLB (1)										1		1								Pos (1)
	France	CLB (2)										2		2								Pos (2)
	Germany	CLB (11)												1								Pos (8)
	Italy	CLB (9)							1			2		2	1	2						Pos (6)
	Switzerland	CLB (18)																				Pos (17)
CC97 (34)	Austria	CLR (6)										3		3								Pos (3)
		CLOG (4)										3		3								Pos (3)
	Belgium	CLI (4)							4			4		4		4						Pos (4)
	Germany	CLI (3)										2		2								Pos (2)
		CLR (2)										1		1								Pos (1)
	Ireland	CLR (2)										2		2								Pos (2)
	Italy	CLF (1)																				Neg
		CLI (1)										1		1								Pos (1)
		CLOG (1)							1			1		1	1							Pos (1)
	Macedonia	CLR (1)																				Neg
	Norway	CLR (2)																				Neg
	Slovenia	CLR (3)													1							Neg
		CLOG (3)						1								1						Neg
	Switzeland	CLR (1)																				Neg
CC705 (44)	Austria	CLC (8)																				Neg
		CLR (1)										1		1								Pos (1)
		CLOG (1)																				Neg
	Belgium	CLC (7)																				Neg
	France	CLC (3)																				Neg
	Germany	CLC (3)																				Neg
	Ireland	CLC (1)																				Neg
		CLOG (1)																				Neg
	Switzerland	CLC (17)								1	1					1						Neg
		CLOG (2)																				Neg
CC20 (8)	Austria	CLF (1)							1			1		1								Pos (1)
	Belgium	CLF (1)																				Neg
	France	CLF (4)															1					Neg
	Italy	CLF (1)												1								Pos (1)
	Slovenia	CLOG (1)																				Pos (1)
CC9 (9)	Austria	CLF (4)										2		2		2						Pos (2)
		CLR (1)										1		1								Pos (1)
	Germany	CLF (2)										1		1		1						Pos (1)
	Italy	CLF (1)																				Neg
	Slovenia	CLOG (1)													1							Neg
Other CC (66)	Austria	CLB (1)																				Pos (1)
		CLC (1)																				Neg
		CLR (6)							1	1	1	1		1	1	1						Pos (1)
		CLOG (5)	1	1								1				2						Pos (1)
	Belgium	CLC (1)																				Neg
		CLI (1)										1		1								Pos (1)
		CLR (1)														1						Neg
		CLOG (2)																				Neg
	France	CLR (1)																				Neg
		CLOG (1)										1		1								Pos (1)
	Germany	CLI (1)										1		1								Pos (1)
		CLR (3)																				Neg
		CLOG (14)			1	1	1					5	1	5	2	1			1		1	Pos (5)
	Ireland	CLR (5)										5		5		1				3		Pos (5)
		CLOG (2)										1		1	1							Pos (1)
	Italy	CLI (1)							1													Neg
		CLOG (5)			1	1	1		1			4		4	2	1			1		1	Pos (4)
	Macedonia	CLR (1)																				Neg
		CLOG (1)										1		1								Pos (1)
	Norway	CLC (1)																				Neg
		CLOG (3)														1						Neg
	Slovenia	CLI (1)																				Neg
		CLR (2)																				Neg
		CLOG (2)													1							Neg
	Switzerland	CLC (2)																				Neg
		CLOG (2)														1						Neg
Total No.			1	1	2	2	2	1	10	2	2	51	1	53	12	20	1	1	2	3	2	88

The abbreviation used in the table for the antibiotics are listed below, and the antibiotics are categorized according to class:

^a^Aminoglycosides: GEN, gentamycin; TOB, tobramycin.

^b^Fluoroquinolones: CIP, ciprofloxacin; LEV, levofloxacin; MOX, moxifloxacin.

^c^Glycopeptides: TEI, teicoplanin.

^d^Lincosamides: CLI, clindamycin.

^e^Macrolides: AZI, azithromycin; ERY, erythromycin.

^f^Penicillins: AMP, ampicillin; OXA, oxacillin; PEN, penicillin.

^g^Tetracyclines: TET, tetracycline.

^h^CH, chloramphenicol; FOS, fosfomycin; FUA, fusic acid; NIT, nitrofurantoin; T/S, trimethoprim/sulfamethoxazole.

The different color gradient is based on the number of positive samples. One is the lowest number of positive samples (light yellow) and the highest number of positive samples is in red.

Among all the ABs, the greatest number of AMR strains were found to be resistant to penicillin/ampicillin, chloramphenicol, clindamycin and tetracycline. There was no AMR observed against most of the tested antibiotics, including vancomycin, trimethoprim/sulfamethoxazole, rifampin, synercid, meropenem, linezolid, imipenem, daptomycin, and ertapenem. Interestingly, no MRSA strains were found.

A total of nine strains (4.3%) were multidrug resistant (MDR). The MDR strains were detected in only four countries: Belgium (*n* = 4, 1.8%), Austria (*n* = 1, 0.5%), Italy (*n* = 3, 1.4%) and Germany (*n* = 1, 0.5%). It is worth noting that the four Belgian strains showed the same pattern of resistance to β-lactams (ampicillin and penicillin), chloramphenicol, and clindamycin. The most resistant strain originated in Italy and showed resistance to β-lactams (ampicillin and penicillin), chloramphenicol, quinolones (ciprofloxacin, levofloxacin, and moxifloxacin), tetracycline, and trimethoprim/sulfamethoxazole.

Supplementary Figure S1 shows the AMR associations found among different ABs (ampicillin, chloramphenicol, clindamycin, penicillin and tetracycline). A strong association was found between the β-lactam ABs (ampicillin and penicillin, *phi* = 1.0; *P* < 0.001). Additionally, a strong association (*phi* = 0.79; *P* < 0.001) was found between clindamycin and chloramphenicol.

To analyze the observed penicillin resistance in more detail, a statistical model was computed to analyze the relationships among the following factors: resistance to penicillin, the most abundant CCs (CC8, CC97, and CC705), countries, and their interactions. For penicillin (*n* = 54), significant interactions (*P* < 0.001 in each case) were observed between penicillin resistance and CCs and between penicillin resistance and countries. Significant values (*P* < 0.001 in each case) were also obtained for the interaction between the CCs and countries, and for individual factors except the CCs (*P* = 0.055). Regarding the CCs, 50% and 14% of the CC97 and CC8 strains, respectively, showed resistance to penicillin. In contrast, CC705 was always sensitive to penicillin. Resistance to penicillin was particularly prominent in Austria, Belgium, Germany, and Ireland, and was absent in Slovenia and Switzerland. An identical loglinear model was also calculated for the genotypic clusters; the most observed CCs were replaced by the three most common CLs (CLB, CLC, and CLR). Significant interactions were found between penicillin resistance and CLs (*P* = 0.014) and between the penicillin resistance and countries (*P* < 0.001). CLC strains were always sensitive to penicillin, whereas 13% of CLB strains and 37% of CLR strains were resistant to penicillin. The distribution of penicillin resistance among the countries was identical to the found in the CCs model. Similar analyses for ABs other than penicillin were not performed due to a lack of sufficient data. In fact, for chloramphenicol and tetracycline, the next most common resistance targets after penicillin, only 20 (9.5%) and 12 (5.7%) of strains demonstrated resistance to these ABs, respectively.

CC705 was not only susceptible to penicillin but also to all other ABs except for one strain that was resistant to azithromycin and erythromycin (both macrolides) and another one that was resistant to chloramphenicol (Table 3). CC97 showed resistance to penicillin, chloramphenicol, and clindamycin. Increased AMR rates, in particular to penicillin/ampicillin and chloramphenicol, were also detected in CC9, CC20, and CC133 (Supplementary Table S4).

### 3.3. Association between MIC and *bla* operon genes

All 54 strains that exhibited phenotypic resistance to penicillin (26% of all strains) showed the simultaneous presence of all *bla* operon genes. In contrast, in 34 strains that were positive for all *bla* genes, the corresponding MIC value was always <0.12 μg/mL. Interestingly, this discrepancy was observed exclusively in CC8/CLB strains with the exception of one strain CC20/GTAT. For 123 trains, the MIC assay and mPCR for *bla* operon genes gave negative results.

## 4. Discussion

### 4.1. Prevalence of *adlb* in European *S. aureus* strains

Previous studies demonstrated that *S. aureus* CC8/CLB is highly contagious (13, 14) and can be detected very specifically by the qPCR assay for *adlb* (22) as also used in the present study. Indeed, with an inclusivity of 97% and exclusivity of 98%, the specificity of this test is very high (22), a fact that was recently confirmed by Gazzola et al. (42). Because of the tight association between CC8/CLB (contagious) and *adlb*, the gene turned out to be a marker for contagiousness and for high prevalence of IMI in dairy herds as shown by Sartori et al. in Swiss and by Maisano et al. in Italian dairy herds (11, 16). Based on the present results we further suggest that high staphylococcal IMI prevalence is also present in Austrian, French, and German dairy herds as *adlb* was regularly observed in the corresponding strains. Indeed, a recent examination of an Austrian and German dairy herd with high IMI prevalence caused by *S. aureus* revealed again the presence of the *adlb* gene. Whether *adlb* is the only staphylococcal marker for contagiousness and high IMI prevalence remains to be elucidated. In fact, the study by Maisano et al. demonstrated that in a small percentage of herds *adlb* was not linked to high staphylococcal IMI prevalence (16).

Interestingly, we detected the *adlb* gene in a German GTI^I^ and a GTR^I^ strain, genotypes that are not part of CLB/CC8. From ongoing studies, we know that the *adlb* gene is located on the staphylococcal cassette chromosome (SCC). As reviewed by Malachowa et al., SCCs may be transmitted among *S. aureus* strains by horizontal gene transfer; hence, the presence of *adlb* gene in GTI^I^ and GTR^I^ strains may be the result of this mechanism, with an *S. aureus* CC8/CLB most likely being the SCC donor (43).

### 4.2. Prevalence of AMR in 10 European countries

In recent years, a general increase in AMR has been reported, and this increase is thought to mainly be due to AB misuse and abuse in agriculture (44, 45). In the worst-case scenario, this AMR could be transmitted to humans, which would aggravate the existing AMR situation faced in human medicine (29). Nevertheless, ABs continue to be a key factor in the treatment of bovine mastitis caused by *S. aureus* (11, 23, 46). Hence, it is vital to use the AB to which an isolate is fully susceptible to guarantee the successful of the therapy. According to our research, despite the large amounts of ABs that have been used to treat bovine IMIs in the past, the AMR status of *S. aureus* isolates from European mastitis cases is promising (47). In fact, all strains were susceptible to most of the 31 ABs tested. AMR was only observed for penicillin (25.6%) ampicillin (24.2%), chloramphenicol (9.5%), clindamycin (4.7%), and tetracyclines (5.7%). Penicillin, chloramphenicol, and tetracycline are ABs that have been widely used in cattle medicine over the past 50 years (48–50). These findings demonstrate and confirm previous observations that the regular use of ABs against *S. aureus* increases the possibility of the emergence of AMR (51, 52). This is in line with our observations that AMR was absent for all ABs whose application, at least in Switzerland, has not been approved for treatment of cattle (50); this is true for all the ABs on the World Health Organization (WHO) reserve list (53, 54), such as daptomycin, linezolid, and fifth-generation cephalosporins. This also holds true for most of the ABs on the WHO watch list (54) including quinolones, carbapenems, fusidic acid (one strain resistant), rifampin, teicoplanin, tobramycin (one strain resistant), and vancomycin; the exceptions were the very limited macrolide (0.9%) and tetracycline (5.7%) resistance. Interestingly, all strains were susceptible to oxacillin and all (except two strains) were susceptible to gentamicin and to trimethoprim/sulfamethoxazole. Obviously, these ABs are still efficient despite their extensive use in cattle medicine. In Switzerland, trimethoprim/sulfamethoxazole is exclusively used as a systemic treatment and is not applied intramammarily (55), so IMI-associated *S. aureus* strains are not in direct contact with this AB, which explain their susceptibility. This contrasts with oxacillin and gentamicin, which have been widely used for the treatment of IMIs in the past 40 years. The minimal AMR prevalence for these AB in bovine *S. aureus* demonstrates that the occurrence of AMRs is not only a matter of frequent use (penicillin and tetracycline). But that it considerably depends on the AB class (aminoglycosides) and even on the properties of the individual compound (oxacillin and penicillin). Considering MRSA, the present study and the one by El Garch co-authors (47) show that MRSA are of no to little concern in the field of bovine mastitis. These observations are in clear contrast to the situation in Swiss human isolates, where the prevalence of MRSA is 6.6% (56). These findings largely suggest that bovine mastitis isolates are not the source of MRSA at the human level.

With a prevalence of 25.6%, penicillin resistance was the most frequently observed type of AMR in our study. This finding aligns with the results of another European study (25.5%) (47) and of an international study (19.4%) that included strains from South America (Argentina, Brazil, and Colombia), South Africa, and the USA (57). Penicillin was introduced for the treatment of bovine mastitis as early as 1945 (58) and is still considered the AB of choice to treat Gram-positive mastitis pathogens (29), which demonstrates its importance in modern medicine.

It is worth noting that resistance to penicillin in bovine *S. aureus* strains can be misreported, as recently shown by Ivanovic et al. (33). Using whole genome sequencing and bioinformatics, the authors showed that the MIC assay, which was also used in the present study, provided the correct results, while analyses conducted using disk diffusion and PCR methods were remarkably flawed (33). Depending on the protocol applied, either too many false negative or false positive results were generated, and false positive results were also generated when the mPCR method was used to assed the *bla* operon genes (*blaI, blaR1, blaZ*). In the case of mPCR, it turned out that the discrepant results were always associated with *S. aureus* CC8/CLB strains. Further genomic analyses of these strains showed that the promoter of the *bla* operon present in the plasmid of the *S. aureus* CC8/CLB strains was inactivated by a 31-bp deletion (33); consequently, the *bla* operon genes that mediate penicillin resistance, were no longer expressed but could be detected by mPCR. The same association, which was explicit for the CC8/CLB strain, between negative MIC values and positive mPCR results was confirmed in the present study. Compared to the previous study (33), however, considerably more strains were evaluated here.

The present study further revealed two more very relevant findings. First, for the three major CCs (CC8, CC97, and CC705) and CLs (CLB, CLC, and CLR), penicillin resistance was highly dependent on the CC and CL. In fact, the CC705 and CLC strains were always susceptible to penicillin whereas penicillin resistance in the CC97 and CLR strains was high, at 50 and 37%, respectively. Penicillin resistance in the CC8 and CLB strains was intermediate, at 14 and 13%, respectively. Importantly, the CC705 and CLC strains were not only susceptible to penicillin but, with two exceptions, also to all other ABs, a property that was not observed for strains in the other CCs and CLs. Second, the prevalence of penicillin resistance is country dependent. Indeed, resistance to penicillin was particularly observed in strains from Austria, Belgium, Germany, and Ireland; however, it was completely absent in strains from Slovenia and Switzerland. It is likely that resistance to other ABs (i.e., chloramphenicol and tetracycline) is also country dependent, although this could not be assessed in the present study because the rate of resistance of other ABs were low and the data set was too small for statistical analyses. Unfortunately, the reason for the difference in penicillin resistance among countries remains unknown and requires further clinical and epidemiological investigations. Nevertheless, our findings demonstrate at least for penicillin, that the prevalence of AMR is country dependent and that caution is required when interpreting results. However, from a statistical and interpretative perspective there are no concerns about analyzing data from multiple-countries as a single entity. In our case, this means that, except for penicillin resistance, the observed prevalence of AMR reflects that at the European level.

## Data availability statement

The original contributions presented in the study are included in the article/Supplementary material, further inquiries can be directed to the corresponding author.

## Author contributions

HG conceived and planned the experiments. GN and LR performed the experimental analyses. AR and HG performed the statistical analyses. All authors discussed the results, and critically revised and approved the final submitted manuscript.
