# Supplementary material for: Bovine Staphylococcus aureus: a European study of contagiousness and antimicrobial resistance
Source: Front Vet Sci. 2023 May 3;10:1154550. doi: 10.3389/fvets.2023.1154550 (PMC10188956; doi:10.3389/fvets.2023.1154550)
Supplement: Supplementary file 1 [file Data_Sheet_1.zip › Tables 1-3 and Figure 1.DOCX]

Supplementary Material

**Table S1**. Overview about the real time quantitative PCR (qPCR) performed including template, primers, and probes based on Sartori et al . 2017

| **Target^1^** | **Primer/Probe name** | **Sequence 5´-3´** | **Amplicon size (bp)** |
| --- | --- | --- | --- |
| *adlb* | Q*adlb*M-S | TGC TAC ATT AGA TTT GCG TAC AGA TA | 95 |
|  | Q*adlb*M-AS | AGA CAT AGC GAC GAT CCT GAT TA |  |
|  | Q*adlb*M-P | CAA CCT CAG CAT AGT AGT TA^2^ |  |
| CDVN | QCDVN-S | CAT CAA CTC TGT TTG CGG TCT TAC^3^ | 130 |
|  | QCDVN-AS | CTT ATT CTC CAA CCA GCC TAA TTG T |  |
|  | QCDVN-P | CTC ATC TGC CTC AGA ATC CAA ACT TGC TC |  |

^1^ *adlb* =gene coding for the adhension-like bovine protein; CDVN = N gene of the canine distemper virus; S = sense primer; AS = antisense primer; P = probe. ^2^ Minor groove binder (MGB) qPCR probe labeled with FAM at the 5′ end and with a confidential no fluorescent quencher at the 3′ end. ^3^Real-time quantitative PCR probe labeled with HEX at the 5′ end and with Black hole quencher1 (BHQ1) at the 3′ end.

**Table S2.** Primers used for amplification of the 3 *bla* operon genes by singleplex melting curve PCR (mPCR) derived from Ivanovic et al., 2023.

| **Gene^1^** | **Primer** | **Sequence 5´-3´** | **Amplicon size (bp)** |
| --- | --- | --- | --- |
| *blaI* | GblaI-S | TCTATGGCTGAATGGGATGTTA | 261 |
|  | GblaI-AS | CATGTCCCCTCCATACAGTTTA |  |
| *blaR1* | GblaR1-S | TATCCATAAGTTTAATTGGGATTC | 461 |
|  | GblaR1-AS | TATCGGCTTCTACTTCATTGT |  |
| *blaZ* | GblaZ-S | AATTCAGATAAGAGATTTGCCTATG | 374 |
|  | GblaZ-AS | CCGAAAGCAGCAGGTGTTGAAGT |  |

^1^*blaI*: gene for repressor of *bla* promotor; *blaR1*: gene for sensor of penicillin G and other β-lactam antibiotics; *blaZ*: β-lactamase gene.

**Table S3**: Range of minimum inhibitory concentration for all the 31 antibiotics molecules analyzed using a **MIC** Panel Type PM32 (Beckman Coulter, Inc., Brea, CA, USA). Supplementary, the resource of the breakpoints were added in an additional column.

|  |  | **MIC Breakpoints (µg/L)** | | |  |
| --- | --- | --- | --- | --- | --- |
| **Class of antibiotics** | **Antibiotics** | **S** | **I** | **R** | **Bibliography** |
| Aminoglycosides | Gentamicin | ≤2 |  | >2 | EUCAST, 2022 |
|  | Tobramycin | ≤ 2 |  | > 2 | EUCAST, 2022 |
| Carbapenems | Ertapenem | ≤2 | 4 | ≥ 8 | CLSI M100-S22 |
|  | Imipenem | ≤4 | 8 | ≥16 | CLSI M100-S22 |
|  | Meropenem | ≤4 | 8 | ≥16 | CLSI M100-S22 |
| Cephalosporins | Cefepime | ≤8 | 16 | ≥32 | CLSI M100-S22 |
|  | Cefotaxime | ≤8 | 16-32 | ≥64 | CLSI M100-S22 |
|  | Cefuroxime | ≤8 | 16 | ≥32 | CLSI M100-S22 |
| Fluoroquinolones | Ciprofloxacin | ≤ 0.001 |  | >1 | EUCAST, 2022 |
|  | Levofloxacin | ≤ 0.001 |  | >1 | EUCAST, 2022 |
|  | Moxifloxacin | ≤ 0.25 |  | > 0.25 | EUCAST, 2022 |
| Glycopeptides | Teicoplanin | ≤ 2 |  | > 2 | EUCAST, 2022 |
|  | Vancomycin | ≤2 |  | ≥2 | EUCAST, 2022 |
| Lincosamides | Clindamycim | ≤0.25 |  | >0.25 | EUCAST, 2022 |
| Lipopeptides | Daptomycin | ≤ 1 |  | >1 | EUCAST, 2022 |
| Macrolides | Azithromycin | ≤ 2 |  | >2 | EUCAST, 2022 |
|  | Erythromycin | ≤1 |  | >2 | EUCAST, 2022 |
| Oxazolidinone | Linezolid | ≤ 4 |  | >4 | EUCAST, 2022 |
| Penicillins | Ampicillin | ≤0.25 |  | ≥0.5 | CLSI M100-S22 |
|  | Oxacillin |  |  | ≥2 | EUCAST, 2022 |
|  | Penicillin | ≤0.125 |  | >0.125 | EUCAST, 2022 |
| Phenicols | Chloramphenicol | ≤ 8 |  | >8 | EUCAST, 2022 |
| Phosphonic | Fosfomycin | ≤ 32 |  | > 32 | EUCAST, 2022 |
| Streptogramine | Synercid | ≤1 | 2 | ≥4 | CLSI, M100-S22 |
| Tetracyclines | Tetracycline | ≤1 |  | ≥2 | EUCAST, 2022 |
| Combinations | Amoxicillin/ Clavulanate | ≤4/2 |  | ≥8/4 | CLSI M100-S22 |
|  | Trimethoprim/ Sulfamethoxazole | ≤ 2/38 |  | ≥ 4/76 | CLSI M100-S22 |
| Other | Nitrofurantoin | ≤64 |  | ≥64 | EUCAST, 2022 |
|  | Fusidic acid | ≤ 1 |  | > 1 | EUCAST, 2022 |
|  | Rifampin | ≤ 0.06 |  | > 0.06 | EUCAST, 2022 |

**Legend:** MIC, minimum inhibitory concentration; EUCAST 2022, European Committee on Antimicrobial Susceptibility Testing (guidelines 2022); CLSI M100-S22, Clinical and Laboratory Standard Institute (Guidelines M100-S22): R, resistant; I, intermediate; S, susceptible.

**Figure S1**: Correlogram showing the association of the main antibiotics resistance (ampicillin, chloramphenicol, clindamycin, penicillin and tetracycline) computed using the phi coefficient. The color gradient represent the phi value of association from 1 (strong correlation) between -1 (no strong correlation).


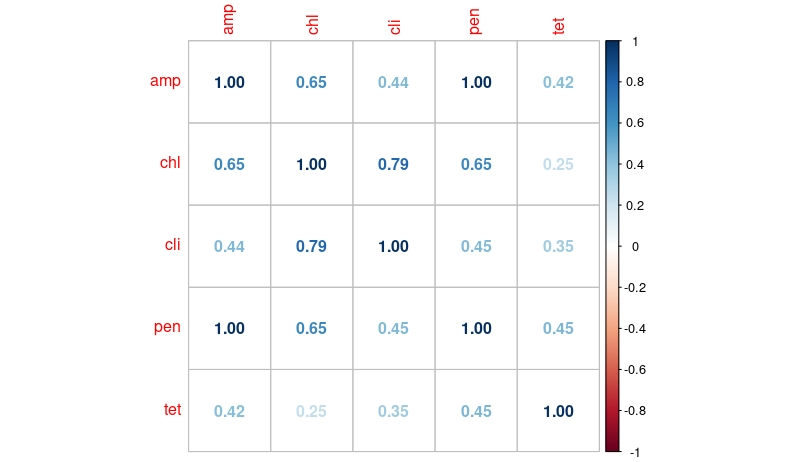


**Legend**: amp: ampicillin, chl: chloramphenicol, cli: clindamycin, pen: pencillin, tet: tetracycline.
